# Qi stagnation and qi deficiency are associated with depression in college students

**DOI:** 10.3389/fpubh.2024.1444237

**Published:** 2024-08-16

**Authors:** Wang Xinzhu, Huang Yuanchun

**Affiliations:** Xichang College, Xichang, China

**Keywords:** depression, qi stagnation, qi deficiency, traditional Chinese medicine, college students

## Abstract

**Objective:**

The current study aims to investigate the correlations between qi stagnation, qi deficiency, and depression levels among college students.

**Method:**

This study investigated 403 college students and measured their levels of depression, qi stagnation, and qi deficiency to analyze the relationship between these three variables. Pearson correlation and linear regression statistical techniques were utilized.

**Results:**

(1) On average, college students reported mild depressive symptoms; (2) college students manifested low levels of qi stagnation and qi deficiency. (3) There exists a strong positive correlation between qi stagnation and qi deficiency; (4) a moderate positive correlation is present between depression and both qi stagnation and qi deficiency among college students. All these results support the mechanism by which qi stagnation and qi deficiency contribute to depression in traditional Chinese medicine theory.

**Conclusion:**

Qi stagnation and qi deficiency are moderately associated with depression levels in college students. It is feasible to use traditional Chinese physical therapy for qi regulation to alleviate depressive symptoms among college students.

## 1 Introduction

The mental health issue of Chinese college students has emerged as a significant social public health concern. Particularly, the detection rate of depression among college students has been on the rise in recent years (1). The overall prevalence of depression among Chinese university students was found to be 28.4% (*n* = 185,787), with 95% CI from 25.7 to 31.2% (2). Depression symptoms have negative effects on the quality of life and academic achievement of college students (3, 4), and impose a significant medical burden on both families and society (5). In recent years, universities in mainland China have not only introduced mental health courses for all students but have also established psychological counseling centers and formally collaborated with hospitals to address the increasing rate of depression among students.

Traditional Chinese medicine (TCM) plays an important role in interpreting and treating depression symptoms in mainland China (6). TCM therapy has unique efficacy in treating mental disorders, including major depression. The effectiveness of acupuncture in treating depression is well documented (7). Meta-analyses have shown that TCM herbal monotherapy is as effective as antidepressants in reducing depression with fewer adverse events than antidepressants (8). However, there is still a lack of research on the mechanisms of depression from a TCM perspective, which significantly limits the role of traditional Chinese medicine in maintaining mental health.

TCM posits that qi pathological status can lead to various diseases in psychology and physiology. Qi is one of the fundamental substances in the human body and is considered to provide energy and power to the body. The balanced state of qi constitutes an essential foundation for mental well-being. The most prevalent types of qi pathological status encompass qi stagnation and qi deficiency. Qi stagnation is characterized by the sluggish, weak, or obstructed flow of qi in specific parts of the body, viscera, or meridians (9). Qi deficiency refers to the lack of qi in one of the viscera or the whole body, so that it cannot provide sufficient energy and power for physical and mental activities (10). According to TCM, there is a mutual influence between qi deficiency and qi stagnation. Qi deficiency leads to weakened qi movement, which easily results in qi stagnation, while qi stagnation can lead to inadequate production of qi, resulting in qi deficiency. For instance, a study investigated 579 outpatients and revealed a positive association between qi deficiency and qi stagnation (*r* = 0.48 in females, and *r* = 0.61 in males) (11).

A few studies have quantitatively examined the association between qi stagnation and mental health. For instance, a study with a sample of 2,108 college students aged 18–25 years demonstrated that qi stagnation due to early life maltreatment can lead to a high level of depressive symptoms among college students (12). A case–control study with a sample of 169 participants aged between 16 and 80 years old found a significant association between qi stagnation and insomnia (13). The close connection between qi deficiency and emotional issues has also been verified through limited research. For example, an experimental study with rat models demonstrated that qi deficiency is related to depression (14). A study revealed that qi deficiency was associated with anxiety disorders in both sexes (11). Generally, lack of energy and vitality, lassitude, and mental and physical fatigue can be attributed to qi deficiency (15). According to TCM, regulating liver-qi and invigorating spleen-stomach-qi is a fundamental approach to treating depression (8).

TCM has been acknowledged by the World Health Organization as one of the potentially valuable complementary medicines (16); however, the association between qi and mental disorders (such as depression) has received scant attention from mental health researchers. The primary objective of this study is to explore the mechanism of depression in college students from a TCM perspective and put forward preventive suggestions based on the research findings. Specifically, this study will investigate the degree of correlation between qi stagnation and qi deficiency and depression among college students and propose potential non-herbal intervention approaches to alleviate depression symptoms among college students. Since depression progresses along a continuum (i.e., from subthreshold depression to major depression) (17), the findings of this study will also be conducive to the TCM treatment of depression symptoms.

The present study proposes two hypotheses as follows:

Hypothesis 1: Qi stagnation is positively associated with depression in college students.

Hypothesis 2: Qi deficiency is positively associated with depression in college students.

## 2 Research method

The current study was conducted in accordance with the Declaration of Helsinki and was approved by the Research Ethics Committee of Xichang College (LG202405). Verbal informed consent was obtained from all participants.

### 2.1 Sampling procedure

The current study is based on cross-sectional data collected from Xichang College, a second-tier university located in Sichuan Province, China. Paper-pencil questionnaires were used, and students were required to provide their student numbers and names for future tracking. The sample consisted of students majoring in business administration, English, primary education, tourism management, and animal science. Students were assured that it was for scientific research and did not pose significant privacy concerns. The survey took place during class breaks from May 20 to 28, 2024. Before the formal survey, the corresponding author coordinated with the teachers in class to help conduct the survey during class breaks.

A total of 452 questionnaires were distributed, and ultimately 403 valid questionnaires were obtained, resulting in an effective rate of 89.16%.

The final valid sample consisted of 97 males (24.07%) and 306 females (75.93%), 315 Han people (78.16%) and 88 individuals from ethnic minorities (21.84%), 306 students from rural registration families (75.93%) and 97 students from urban registration families (24.07%). The age range of the participants was between 17 and 23 years, with a mean age of 19.74 years and a standard deviation of 1.06.

### 2.2 Research instruments

This study utilized six items to assess qi stagnation and six items to measure qi deficiency. These items were developed based on previous TCM studies (10, 18). Participants were instructed to recollect their physical sensations over the past 3 months and respond to 12 items using a 5-point Likert scale ranging from 1 (never) to 5 (often). One of the items measuring qi stagnation stated, “I sigh for no reason.” One of the items assessing qi deficiency stated, “I get tired easily.” The Cronbach alpha coefficients for qi stagnation and qi deficiency were 0.81 and 0.85, respectively, indicating high reliability. High mean scores on the scales indicated high levels of qi stagnation or qi deficiency among the students.

This study employed PHQ-9 to assess depression symptoms, which is the depression module of the Patient Health Questionnaire (PHQ), a self-administered instrument to diagnose common mental disorders (19). One of the nine items reads, “I have little interest or pleasure in doing things.” Participants were instructed to assess their emotional condition during the previous week and respond on a four-point Likert scale with “0” denoting “not at all,” “1” meaning “several days,” “2” indicating “more than half the days,” and “3” meaning “nearly every day.” The Cronbach alpha coefficient of this measurement is 0.84. Higher composite average scores indicate higher levels of depression.

Sleep quality is a control variable in the present study, and it is measured by a single item derived from the study by Snyder et al. (20). This study defines good sleep in the following manner: it is easy to fall asleep when lying down on the bed, rarely being awakened during the night, and feeling refreshed and restored upon waking in the morning. Participants were instructed to retrospectively assess their sleep quality over the past month and evaluate it using a 5-point scale based on the criteria of good sleep. In this 5-point scale, “1” denoted “very poor,” while “5” signified “excellent.”

Physical exercise is another control variable, measured with a single item adopted from Milton et al. (21)‘s study. The present study characterizes healthy physical activity in the following manner: Each session should last a minimum of 20 min and result in at least mild perspiration or breathlessness, and the forms of activities may include brisk walking, table tennis, basketball, badminton, football, running, skipping, dancing, swimming, etc. Participants were asked to retrospectively assess their exercise activity over the past month and evaluate it using a 5-point scale, based on the criteria of healthy physical exercise. In this 5-point scale, “1″ means “Less than once a week,” “2″ means “1–2 times a week,” “3″ denotes “3–4 times a week,” “4″ indicates “5–6 times a week,” and “5″ means “almost every day.”

Diet quality was also assessed as a control variable, using a single item adapted from a previous study (22). The study defines a healthy diet in this manner: in addition to staple foods, the daily diet should also include soy products (such as tofu), fruits, milk or other dairy products, eggs, meats (such as pork, chicken, or beef), and seafood (such as fish and shrimp). Participants were asked to rate their diet quality over the past 3 months according to the above definition of a healthy diet, using a five-point scale with “1” representing “very poor” and “5” indicating “very good.”

Additionally, considering the significant impact of academic pressure on depression levels, this study also included academic stress as a control variable. Two items assessing academic pressure were adapted from Bedewy and Gabriel (23)‘s research. One item required participants to rate their workload in class during the semester using a five-point scale ranging from “1″ for “very light” to “5″ for “very heavy.” The other item asked participants to rate their workload after class during the semester on a five-point scale, with “1″ indicating “very little” and “5″ indicating “quite a lot.” High composite average scores of these two items indicated greater academic pressure. The Cronbach's reliability coefficient for academic pressure in this study was 0.84.

Finally, key personal information of college students was also gathered, including gender (male = 0, female = 1), family registration area (rural = 0, urban = 1), ethnicity (Han = 1, minorities = 1), and age.

## 3 Results

### 3.1 Descriptive results

Descriptive statistics of some continuous variables are presented in Table 1.

**Table 1:** Descriptive statistics for key variables.

	Min	Max	*M*	SD
DE	0.00	2.56	0.63	0.46
QS	1.00	4.50	1.98	0.73
QD	1.00	5.00	2.44	0.89
PE	1.00	5.00	2.03	0.94
SQ	1.00	5.00	3.17	0.97
DQ	1.00	5.00	3.41	0.80
AP	1.00	5.00	3.04	0.66
FES	1.00	5.00	2.86	0.57

DE, depression; QS, qi stagnation; QD, qi deficiency; PE, physical exercise; SQ, sleep quality; DQ, diet quality; AP, academic pressure; FES, Family economic status; FRA, family registration area; Min, Minimum; Max, Maximum; M, Mean; SD, standard deviation.

In this study, the mean depression score was 0.63 on a 4-point scale of 0–3, resulting in a composite sum score of 5.67 (0.63 multiplied by 9). According to Kroenke et al. (24), composite sum scores falling within the range of [5, 9] indicate mild levels of depression. Therefore, the findings suggest that college students, as a whole, experienced mild levels of depression.

On the 5-point scale of 1–5, composite average scores falling in the range of 1.81–2.61 indicate a low level, while scores falling within the interval of 2.62–3.42 indicate a moderate level (25). In this study, the mean values for qi stagnation and qi deficiency were 1.98 and 2.44, respectively. Therefore, overall, college students in this study reported low levels of qi stagnation and qi deficiency symptoms.

The mean values of sleep quality, diet quality, and physical exercise were 3.17, 3.41, and 2.03, respectively, on a five-point scale from 1 to 5. According to Alkharusi (25), these mean scores indicated that college students reported moderate levels of sleep quality and diet quality, as well as low levels of physical exercise.

On the 5-point scale of 1–5, the composite mean values for academic pressure and family economic status are 3.04 and 2.86, respectively. According to Alkharusi (25), these mean values suggest that college students in the current study experience a moderate level of academic pressure and perceive their family's economic status as being at a moderate level.

**Figure 1:**
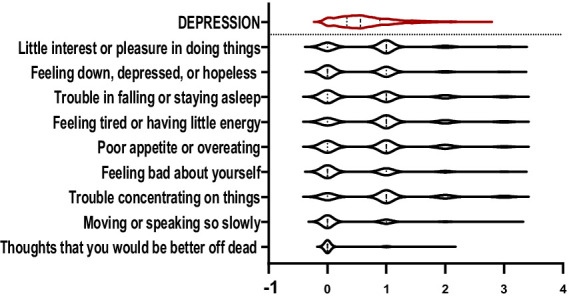
The description of depression and its items.

**Figure 2:**
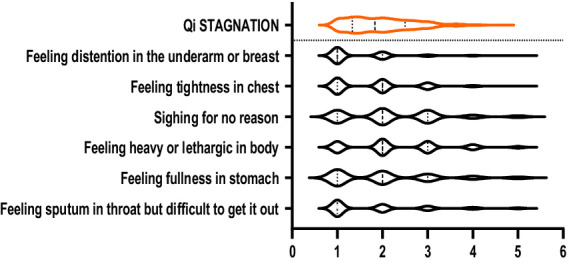
The description of Qi stagnation and its items.

**Figure 3:**
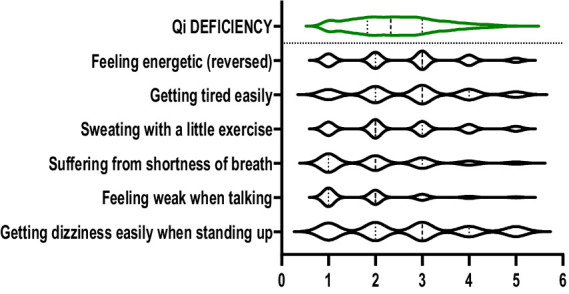
The description of Qi deficiency and its itmes.

**Figure 4:**
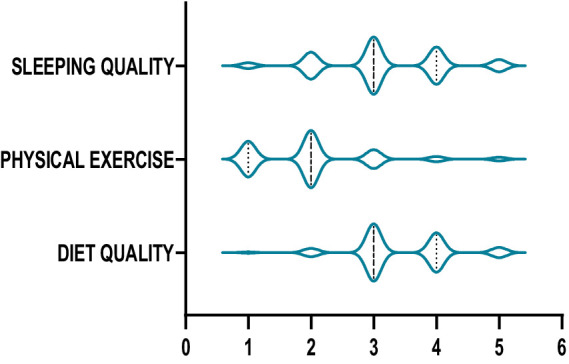
The description of life style.

**Figure 5:**
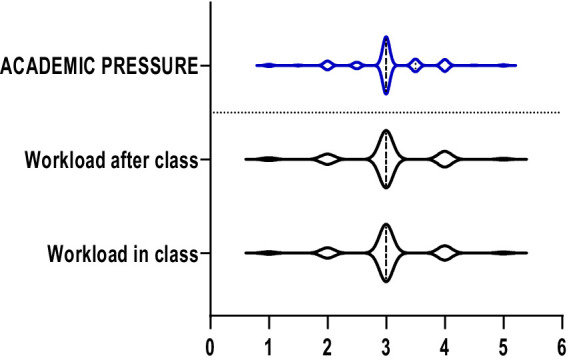
The description of academic pressure and its items.

**Figure 6:**
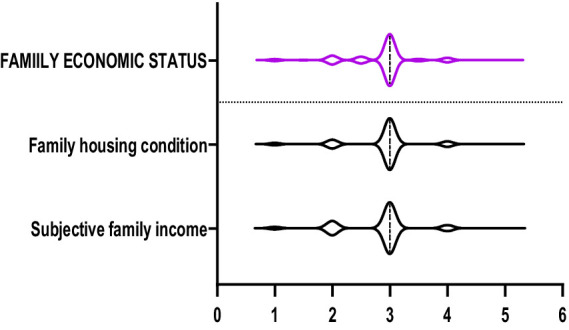
The description of family economic status and its items.

The violin plots in Figures 1–6 display the score distribution for all measured items and the composite mean distribution for each variable, respectively.

### 3.2 The results of correlational analysis

The current study investigates the magnitude of simple correlations among variables, and the Pearson correlation coefficients are presented in Table 2.

**Table 2:** Correlation coefficients between variables.

	DE	QS	QD	PE	SQ	DQ	AP
DE	1						
QS	0.618^**^	1					
QD	0.633^**^	0.776^**^	1				
PE	−0.075	−0.131^**^	−0.218^**^	1			
SQ	−0.349^**^	−0.250^**^	−0.239^**^	0.113^*^	1		
DQ	−0.272^**^	−0.230^**^	−0.241^**^	0.070	0.298^**^	1	
AP	0.103^*^	0.142^**^	0.202^**^	−0.090	−0.033	−0.010	1
FES	−0.088	−0.030	−0.049	−0.007	0.141^**^	0.106^*^	0.006

^**^Denotes significance at the 0.01 level; ^*^indicates significance at the 0.05 level. DE, depression; QS, qi stagnation; QD, qi deficiency; PE, physical exercise; SQ, sleep quality; DQ, diet quality; AP, academic pressure; FES, Family economic status; FRA, family registration area; Min, Minimum; Max, Maximum; M, Mean; SD, standard deviation.

The results presented in Table 1 revealed a positive correlation between qi stagnation and depression (*r* = 0.618, *p* < 0.01), as well as between qi deficiency and depression (*r* = 0.633, *p* < 0.01). The correlation between physical activity and depression did not reach statistical significance (*r* = −0.075, *p* > 0.05), while sleep quality showed a negative correlation with depression (*r* = −0.349, *p* < 0.01). Additionally, diet quality was negatively correlated with depression (*r* = −0.272, *p* < 0.01), and academic pressure was positively correlated with depression (*r* = 0.103, *p* < 0.05). Family economic status showed a non-significant association with depression (*r* = −0.088, *p* > 0.05).

**Figure 7:**
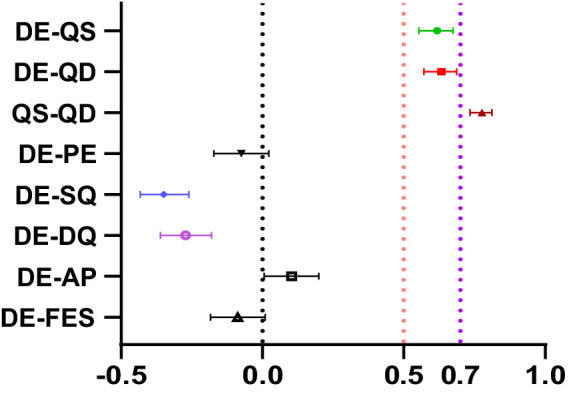
The main correlation coefficients bootstrapped 1,000 times with a 95% confidence interval. DE, depression; QS, qi stagnation; QD, qi deficiency; PE, physical exercise; SQ, sleep quality; DQ, diet quality; AP, academic pressure; FES, Family economic status; FRA, family registration area; Min, Minimum; Max, Maximum; M, Mean; SD, standard deviation.

The sample was bootstrapped 1,000 times, and 95% confidence intervals for the main correlation coefficients were obtained (refer to Figure 7).

According to Schober et al. (26), the absolute magnitude of the observed correlation coefficient is categorized as follows: 0.00–0.10 represents negligible correlation, 0.10–0.39 represents weak correlation, 0.40–0.69 represents moderate correlation, and 0.70–0.89 represents strong correlation. In this study, the confidence intervals for the correlation coefficients between depression and qi stagnation, and between depression and qi deficiency were [0.554, 0.675] and [0.571, 0.688], respectively; while the confidence interval for the correlation coefficient between qi stagnation and qi deficiency was [0.734, 0.812]. Therefore, there was a moderate correlation between depression and qi stagnation as well as between depression and qi deficiency; whereas there was a strong correlation between qi stagnation and qi deficiency.

In addition, the confidence interval of the correlation coefficient between depression and sleep quality was [−0.432, −0.260], indicating a weak to moderate relationship; while that between depression and diet quality was [−0.361, −0.180], suggesting a weak association between diet quality and depression.

Finally, the correlations between depression and physical exercise, academic pressure, and family economic status are all at a negligible level.

### 3.3 Results of regression analysis

Depression is considered the dependent variable, with qi stagnation and qi deficiency as independent variables, while also including control variables in the model. The regression results are presented in Table 3.

**Table 3:** Regression results of DE on QS and QD.

	*B*	SE	*β*	*t*	Tolerance	VIF
(Constant)	0.943	0.353		2.674^**^		
QS	0.185	0.036	0.290	5.107^**^	0.385	2.594
QD	0.204	0.030	0.389	6.708^**^	0.370	2.704
PE	0.016	0.018	0.033	0.871	0.891	1.123
SQ	−0.078	0.018	−0.162	−4.237^**^	0.848	1.179
DQ	−0.048	0.022	−0.083	−2.157^*^	0.850	1.176
AP	0.006	0.026	0.009	0.243	0.940	1.064
FES	−0.003	0.030	−0.004	−0.099	0.908	1.102
FRA	−0.018	0.040	−0.017	−0.464	0.921	1.086
Gender	−0.167	0.041	0.154	4.090^**^	0.878	1.139
Ethnicity	0.073	0.042	0.065	1.732	0.878	1.139
Age	−0.030	0.016	−0.068	−1.870	0.941	1.063
	*F* = 37.523^**^		*Adj.R*^2^ = 0.500			

^**^Denotes significance at the 0.01 level; ^*^indicates significance at the 0.05 level. DE, depression; QS, qi stagnation; QD, qi deficiency; PE, physical exercise; SQ, sleep quality; DQ, diet quality; AP, academic pressure; FES, Family economic status; FRA, family registration area; Min, Minimum; Max, Maximum; M, Mean; SD, standard deviation.

In Table 3, the variance inflation factor (VIF) is significantly below 5, indicating the absence of any apparent collinearity issues among the aforementioned variables (27).

It is evident from Table 3 that qi stagnation has a significant impact on depression (*β* = 0.290, *p* < 0.01). Additionally, qi deficiency also demonstrates a significant effect on depression (*β* = 0.389, *p* < 0.01).

In the realm of lifestyle factors, it was found that diet quality had a significant impact on depression (*β* = −0.083, *p* < 0.05), while physical exercise did not exhibit a significant effect (*β* = 0.033, *p* > 0.05). On the other hand, sleep quality demonstrated a significant effect (*β* = −0.162, *p* < 0.01).

Family economic status (*β* = −0.004, *p* > 0.05) and academic pressure (*β* = 0.009, *p* > 0.05) had no significant effect on depression.

Among the individual characteristics of age, gender, family registration area, and ethnicity, only gender demonstrated a significant effect on depression (*β* = 0.154, *p* < 0.01).

## 4 Discussion

### 4.1 Descriptive statistical results

The study demonstrates that, on average, college students exhibit mild symptoms of depression, which aligns with the findings in the study by Du Na et al. (28). Du Na et al. (28) also used the PHQ-9 with a 4-point Likert scale ranging from 0 to 3 to assess college students’ depression. Their results revealed a composite mean score of 0.701 on the depression scale (with a composite sum score of 6.38), indicating that, as a whole, college students experienced mild depressive symptoms.

In this study, college students reported low levels of qi stagnation symptoms, with a mean score of 1.98 falling between “never” and “rarely” on the 5-point scale, closely approaching “rarely.” Additionally, college students exhibited relatively low levels of qi deficient symptoms, averaging at 2.44, which falls within the range of “rarely” to “occasionally” on a 5-point scale. It is evident that compared to qi stagnation symptoms, college students reported more symptoms of qi deficiency.

The lifestyle of the college students in this study was sub-healthy. Firstly, their diet quality and sleep quality were moderately healthy, consistent with previous research such as Xu Chunyan et al. (29), which reported sub-healthy diet quality and sleep quality among Chinese college students. Secondly, college students reported a low level of physical exercise with merely 1–2 times physical activity per week, and each session lasts more than 20 min. Such a finding aligns with the result in the study by Le Junchao and Guan Yuan (30), revealing that college students engage in sports 1–2 times per week.

The college students in this study perceive their family's economic status to be at a moderate level. This is primarily attributed to the fact that Xichang college is a second-tier university, and the majority of its students come from lower socioeconomic backgrounds. For instance, 75.93% of the students in this study come from rural-registered families.

College students in this study reported moderate levels of academic pressure, which is consistent with the actual situation at Xichang college. As a second-tier university, its academic requirements are not overly rigorous, and the academic competition among students is relatively low.

### 4.2 The magnitude of correlations in simple correlational analysis

The correlation analysis in this study revealed a strong positive correlation between qi stagnation and qi deficiency. Such a finding is similar to that of Kondo et al. (11), who observed a moderate to strong positive correlation between qi stagnation and qi deficiency in psychological outpatients (*r* = 0.48 in females, and *r* = 0.61 in males).

The Pearson correlation analysis revealed a moderate correlation between qi stagnation and depression, which lends support for Hypothesis 1. Such a finding is in line with the findings in the study by Huang Huiyuan et al. (12), which reported that qi stagnation was moderately associated with depression (*r* = 0.556). There is a moderate positive correlation between qi-deficiency and depression, supporting Hypothesis 2 very well. This finding is consistent with the findings in the study by Zhang Xiaocong et al. (31) who observed a moderate positive correlation (*r* = 0.41) between qi deficiency and depression among college students.

Regarding control variables, we observed a weak to moderate inverse relationship between depression and sleep quality, which aligns with the findings of Zhang Xin et al. (32) who identified a moderate correlation (*r* = 0.41) between sleep quality and depression levels in Chinese students. In addition, the present study revealed a weak negative association between depression and diet quality, as found in a literature review revealing a weak to moderate negative association between diet quality and depression in diverse population (33). Lastly, there were non-significant negative correlations between physical activity and family economic status with depression, which contrasts with previous studies that found significant negative correlations [e.g., (34, 35)]. This discrepancy may be attributed to the limited number of items used in this study to assess physical activity and family economic status, leading to substantial measurement errors.

### 4.3 The effects of qi stagnation and qi deficiency on depression in regression analysis

In the regression analysis, we controlled for diet quality, sleep quality, physical exercise, academic pressure, family economic status, and demographic characteristics. The regression results indicated that both qi stagnation and qi deficiency were significant positive predictors of depression level (refer to Table 3; Figure 7); these results also substantiate research hypotheses 1 and 2. These findings are in line with previous research conclusions. For instance, a study comparing adults with and without qi stagnation found higher levels of anxiety and depression among those with qi stagnation (36). Li Xiaojuan et al. (14) exposed the rats to different durations of chronic unpredictable mild stress (CUMS), and found that the rats exposed to 6-week CUMS procedure exhibited significantly similar traits to the phenotypes of LQSSDS (meaning qi stagnation in the liver, and qi deficiency in spleen simultaneously) and depression. According to the TCM theory, the normal state of qi is fundamental to maintaining a healthy mental state; when qi is in a pathological state, mental functions will be hurt, and cognitive decline and emotional disturbances then ensue.

This study further revealed that the effect of qi deficiency on depression was stronger than that of qi stagnation, suggesting that qi deficiency exerts a greater effect on depressive symptoms than qi stagnation in college students. The reason lies in the fact that qi deficiency symptoms were more common among college students compared to qi stagnation symptoms (see Table 1). Insufficient qi is not able to supply adequate nourishment and energy for mental functions. Consequently, qi deficiency has a more significant impact on depression than qi stagnation. In clinical practice of TCM, heart-spleen deficiency (qi deficiency in both heart and spleen) is a common type of depressive disorders, and the patients in this type have more prominent symptoms of qi deficiency than symptoms of qi stagnation (37). A system review showed that among the top three herb formulas for patients with sleep disorder and major depressive disorder during 2007–2011 in Taiwan, Jia-Wei-Xiao-Yao-San came first (38). Jia-Wei-Xiao-Yao-San has been traditionally used to tonify the qi in the spleen and stomach. It is evident that qi deficiency significantly contributes to the development of depression symptoms.

### 4.4 The effects of control variables on depression in regression analysis

Among the control variables, sleep quality is correlated with depression levels, consistent with previous research findings. For instance, a systematic review found that college students with high levels of depression tend to have poor sleep quality (39). Poor sleep quality is, in fact, a significant symptom of depression.

In this study, diet quality was associated with depression, which is consistent with previous research. For instance, a systematic review of 24 independent cohorts (totaling 1,959,217 person-years) showed higher diet quality is associated with a lower risk for the onset of depressive symptoms (40). According to TCM theory, diet quality is closely associated with the qi in spleen and stomach, and unhealthy diet can lead to inadequate production of qi in spleen and stomach, resulting in qi deficiency and impacting mental functions.

Ultimately, this study revealed that gender was associated with depression levels, with female students exhibiting higher levels of depression than male ones. Such a finding is consistent with previous research [e.g., (41)], possibly due to the significantly higher levels of qi stagnation and qi deficiency in females compared to males (see Appendix 1).

### 4.5 Limitations of the present study

In this study, cross-sectional data were employed to explore the association between qi stagnation, qi deficiency, and depression symptoms in college students. All hypotheses in this study are well supported, suggesting that this study possesses good internal validity. Nevertheless, this study has the following three drawbacks.

Firstly, the utilization of cross-sectional data restricts the capability to establish causation and only allows for correlation conclusions. Secondly, the small and non-representative sample size, with 75.93% of college students being from rural households and 75.93% being women, limits the external validity of the study. Finally, utilizing a single measure for assessing sleep quality, diet quality, and physical activity may introduce substantial measurement errors and compromise the study's reliability. In future research, we plan to expand our sample size properly, conduct follow-up surveys on participants, and utilize cross-lagged analysis to further explore the impact of qi stagnation and qi deficiency on depression.

## 5 The implications of the present study

The current study holds both theoretical and practical significance. On the theoretical side, this study offers a distinct perspective on explaining the mechanism of depression symptoms. According to TCM theory, qi is considered as one of the essential material bases for normal mental functions. When qi is in deficiency, it is not able to supply adequate nourishment and energy for mental activities, leading to a decline in mental functions. Qi stagnation refers to slow and idle flow of qi, which can also result in inadequate nourishment for mental activity. 《Simple question. On the theory of pain (素问・举痛论)》 posits that various diseases arise from qi pathological states, indicating that qi stagnation and qi deficiency can lead to numerous kinds of diseases. To summarize, the current study offers support for the TCM theory that depressive symptoms are attributed to qi stagnation and qi deficiency.

In terms of preventing depression, this study suggests that in addition to psychological counseling, using TCM to alleviate symptoms of qi stagnation and qi deficiency can be beneficial for helping college students prevent the onset and progression of depression symptoms. Common health care methods including massage (42), Guasha (scraping) (43), acupuncture (44), and auricular acupressure (45) all can effectively replenish qi and regulate its flow. The integration of TCM health care techniques with psychological counseling can more effectively aid college students in preventing severe depression.

Additionally, according to TCM, a healthy lifestyle (comprising appropriate exercise, a healthy diet, and good sleep) is beneficial for regulating the state of qi (46). Hence, it is indispensable for college students to adopt a healthy lifestyle to prevent and ameliorate depressive symptoms.

## 6 Conclusion

The findings from this study lend support to the viewpoints in TCM that the healthy state of qi is essential for mental health. Specifically, we revealed that both qi stagnation and qi deficiency are strongly and positively associated with depression symptoms in college students.

The conclusion of this study indicates that the application of certain TCM physiotherapy techniques for regulating and replenishing qi in the body can alleviate depression symptoms among college students. This study is of a correlational rather than causal nature, and we will follow up on these participants in the future to further analyze the connection between qi stagnation, qi deficiency and depressive symptoms.

## Data availability statement

The raw data supporting the conclusions of this article will be made available by the authors, without undue reservation.

## Ethics statement

The studies involving humans were approved by Teacher Education Division of Academic Committee in Xichang University. The studies were conducted in accordance with the local legislation and institutional requirements. The participants provided their written informed consent to participate in this study.

## Author contributions

WX: Conceptualization, Methodology, Writing – original draft. HY: Conceptualization, Investigation, Methodology, Writing – review & editing.

## Appendix 1

Test for the difference between Qi deficiency and Qi stagnation in gender

There is a significant difference in qi stagnation between the sexes, with females (*M* = 2.06, SD = 0.74) exhibiting significantly higher levels than males (*M =* 1.74, *SD* = 0.63; *t* = −3.81, *p* < 0.01).

There is a significant difference in qi deficiency between the sexes, with females (*M* = 2.54, *SD* = 0.88) exhibiting significantly higher levels than males (*M* = 2.14, *SD* = 0.84; *t* = −3.90, *p* < 0.01).
